# Transport Activity of the Sodium Bicarbonate Cotransporter NBCe1 Is Enhanced by Different Isoforms of Carbonic Anhydrase

**DOI:** 10.1371/journal.pone.0027167

**Published:** 2011-11-04

**Authors:** Christina Schueler, Holger M. Becker, Robert McKenna, Joachim W. Deitmer

**Affiliations:** 1 Abteilung für Allgemeine Zoologie, FB Biologie, TU Kaiserslautern, Kaiserslautern, Germany; 2 AG Zoologie/Membrantransport, FB Biologie, TU Kaiserslautern, Kaiserslautern, Germany; 3 Department of Biochemistry and Molecular Biology, College of Medicine, University of Florida, Gainesville, Florida, United States of America; Emory University, United States of America

## Abstract

Transport metabolons have been discussed between carbonic anhydrase II (CAII) and several membrane transporters. We have now studied different CA isoforms, expressed in *Xenopus* oocytes alone and together with the electrogenic sodium bicarbonate cotransporter 1 (NBCe1), to determine their catalytic activity and their ability to enhance NBCe1 transport activity. pH measurements in intact oocytes indicated similar activity of CAI, CAII and CAIII, while *in vitro* CAIII had no measurable activity and CAI only 30% of the activity of CAII. All three CA isoforms increased transport activity of NBCe1, as measured by the transport current and the rate of intracellular sodium rise in oocytes. Two CAII mutants, altered in their intramolecular proton pathway, CAII-H64A and CAII-Y7F, showed significant catalytic activity and also enhanced NBCe1 transport activity. The effect of CAI, CAII, and CAII mutants on NBCe1 activity could be reversed by blocking CA activity with ethoxyzolamide (EZA, 10 µM), while the effect of the less EZA-sensitive CAIII was not reversed. Our results indicate that different CA isoforms and mutants, even if they show little enzymatic activity *in vitro*, may display significant catalytic activity in intact cells, and that the ability of CA to enhance NBCe1 transport appears to depend primarily on its catalytic activity.

## Introduction

Maintenance of H^+^ homeostasis is important for all cell types, because protons are involved in many processes of metabolism and cellular function. The regulation of intra- and extracellular proton concentration is due to proton buffer capacity, which includes the CO_2_/HCO_3_^−^-dependent and intrinsic buffer capacity, and the active transport of acid-/base-equivalents across cell membranes. Cells possess a number of ion transporters, which are involved in the active transport of H^+^ or HCO_3_^−^, like sodium hydrogen exchangers (NHE) [1] or the solute carrier family 4 of bicarbonate transporters (SLC4) [2], [3]. For several families of acid-/base-coupled transporters an interaction of the transporter with carbonic anhydrase II (CAII) has been shown, resulting in an enhanced transport activity of the acid-/base-transporters [4]–[10].

CAII regulates the concentration of the substrate of the sodium bicarbonate cotransporter 1 (NBCe1) by catalyzing the hydration of CO_2_ and the dehydration of HCO_3_^−^. A functional transport metabolon between the two proteins has been suggested by several groups [7], [11]. We have previously shown an increase in transport activity of NBCe1 by CAII in *Xenopus* oocytes as determined by an increased membrane current, rate of rise of intracellular sodium concentration and membrane conductance [12]. In contrast, other groups could not find evidence for an interaction of CAII and NBCe1 by determining the effect on membrane conductance of NBCe1-expressing oocytes after an injection of CAII-protein [13] or determining change of membrane current of NBCe1+CAII-coexpressing oocytes [14].

We have now investigated the interaction of NBCe1 and CA by the use of two additional intracellular isoforms of CA, CAI and CAIII, and several CAII mutants with altered proton transfer. The isoforms CAI and CAIII are known to exhibit different catalytic activities than CAII - CAIII about 0.3% of CAII activity [15]–[17] and CAI about 15% [18], [19]. Replacement of the histidine at position 64, which plays a central role for the proton shuttle in the catalytically highly active CAII, with alanine (CAII-H64A), resulted in a more than 10-fold decrease of the turnover number in the absence of buffers *in vitro* [20], [21]. Different amino acids (Tyr7, Asn62, His64, Asn67, Thr199 and Thr200), located in the active site of CAII, are involved in the stabilization of a network of water molecules [22], [23]. Mutation of CAII tyrosine at position 7 into a phenylalanine (CAII-Y7F) has been shown to lead to a seven-fold increase of the proton transfer rate in direction of dehydration [24], due to a less branched water wire [25]. An increase in catalytic activity of this mutant could, however, not be detected [25], [26].

We have employed these CAII mutants, in addition to the two intracellular isoforms CAI and CAIII, to explore the role of catalytic activity and intramolecular proton shuttling of CA for NBCe1 transport activity. Our results indicate that the interaction of different CA-isoforms with NBCe1 solely depends on the catalytic activity and appears to be independent of the intramolecular proton shuttle in CA. Furthermore, the catalytic activity of CAI, CAIII and the mutant CAII-H64A is increased or restored in intact oocytes.

## Materials and Methods

### Constructs, oocytes, and injection of cRNA and carbonic anhydrase

The CAI-cDNA (CAI-WT) was purchased from OriGene Technologies (Rockville, USA). CAII-cDNA (CAII-WT) was kindly provided by Dr. Reinhart Reithmeier [27]. The cDNA of the catalytically inactive mutant CAII-V143Y was a gift from Dr. Carol Fierke (Ann Arbor, USA) [28], [29]. The cDNA of CAI, CAII, CAII-V143Y, CAII-H64A [21], [30], CAII-Y7F [24], [25] and CAIII was subcloned into the oocyte expression vector pGemHeJuel, which contains the 5′ and the 3′ untranscribed regions of the *Xenopus* β-globin. The human NBCe1 cDNA (hkNBCe1) was cloned in the oocyte expression vector pGH19. Plasmid DNA was linearized with NotI and transcribed *in vitro* with T7 RNA-Polymerase in the presence of the cap analogon m7G(5′)ppp(5′)G (mMessage mMachine, Ambion Inc., USA) to produce a capped RNA transcript. The cRNA was purified with the RNeasy MinElute Cleanup Kit (Qiagen GmbH, Hilden, Germany) and stored at −80°C in DEPC-H_2_O. *Xenopus laevis* females were purchased from Xenopus Express (Vernassal, France). Frogs were anesthetized with 1 g/l of 3-aminobenzoic acid ethylester (MS-222; Sigma-Aldrich, Taufkirchen, Germany), added to their bath, rendered hypothermic and segments of ovarian lobules were surgically removed. The procedure was approved by the Landesuntersuchungsamt Rheinland-Pfalz (Koblenz, Germany; 23 177-07/A07-2-003 §6).

Oocytes were isolated and singularized by collagenase treatment (Collagenase A, Roche, Mannheim, Germany) in Ca^2+^-free oocyte saline at 28°C for 2 h. The singularized oocytes were left overnight in an incubator at 18°C in Ca^2+^-containing oocyte saline (pH 7.8) to recover. The oocyte saline had the following composition (in mM): NaCl, 82.5; KCl, 2.5; CaCl_2_, 1; MgCl_2_, 1; Na_2_HPO_4_, 1; HEPES, 5, titrated with NaOH to pH 7.4. Oocytes of the stages V and VI were selected and injected with 13.8 ng of NBCe1-cRNA using glass micropipettes and a microinjection device (Nanoliter 2000, World Precision Instruments, Berlin, Germany). CAI and CAII were either injected as protein or coexpressed with the NBCe1. For injection of protein, different amounts of CAI (0–200 ng), isolated from human erythrocytes (Sigma-Aldrich), or 50 ng of CAII, isolated from bovine erythrocytes (Sigma-Aldrich), dissolved in 27.6 nl DEPC-H_2_O, were injected 12–24 h before oocytes were used for electrophysiological measurements. For coexpression of CAI, CAII, CAIII or CAII-mutants, respectively, 11.5 ng CA-cRNA was injected either alone or together with the NBCe1-cRNA.

### Intracellular pH and Na^+^ measurements

For measurement of intracellular pH (pH_i_) and membrane potential, double-, and for intracellular Na^+^ (Na^+^_i_), single-barreled microelectrodes were used; the manufacture and application have been described in detail previously [31], [32]. Briefly, for double-barreled microelectrodes, two borosilicate glass capillaries of 1.0 and 1.5 mm in diameter were twisted together and pulled to a micropipette. The ion-selective barrel was silanized with a drop of 5% tri-N-butylchlorsilane in 99.9% pure carbon tetrachloride, backfilled into the tip. The micropipette was baked for 4.5 min at 450°C on a hot plate. H^+^-sensitive cocktail (Fluka 95291, Fluka, Buchs, Switzerland) was backfilled into the tip of the silanized ion-selective barrel and filled up with 0.1 M Na-citrate, pH 6.0. The reference barrel was filled with 3 M KCl. Calibration of the pH-sensitive microelectrodes was carried out in oocyte salines by changing the pH by 0.6 units. Electrodes were accepted for use in the experiments, when their response exceeded 50 mV per unit change in pH. For Na^+^-sensitive microelectrodes, a 1.5 mm borosilicate glass capillary was silanized as described above and backfilled with Na^+^- sensitive cocktail, made of 10 wt% sodium ionophore VI (Fluka 71739), 89.5 wt% 2-nitrophenyl octyl ether and 0.5 wt% sodium tetraphenylborate. The pipette was filled up with a solution containing 100 mM NaCl and 10 mM MOPS buffer, pH 7.0. Calibration was carried out in oocyte saline with Na^+^ concentrations of 5, 10, 15 and 84.5 mM. Na^+^-selective electrodes responded for a tenfold change in the Na^+^ concentration (85 to 8.5 mM) with 58 mV, when Na^+^ was replaced by NMDG or Li^+^, and with 54 mV, when Na^+^ was replaced by K^+^. As described previously [33], optimal changes in ion concentration were detected when the electrode was located near the inner surface of the plasma membrane. All experiments were carried out at room temperature. Oocyte saline titrated with N-methyl-D-glucamine to pH 7.4 or 7.0 was used. The 24 mM bicarbonate-buffered saline (pH 7.4) contained (in mM): NaCl, 58.5; KCl, 2.5; CaCl_2_, 1; MgCl_2_, 1; Na_2_HPO_4_, 1; NaHCO_3_, 24, aerated with 5% CO_2_ and HEPES, 5, to stabilize the pH. In 10 mM (pH 7.0) and 77 mM (pH 7.9) bicarbonate-buffered saline, NaCl was replaced by an equimolar amount of NaHCO_3_.

The measurements of pH_i_ were stored digitally using homemade PC software based on the program LabView (National Instruments Germany GmbH, München, Germany) and were routinely converted into intracellular H^+^ concentration [H^+^]_i_. Thus, changes in the [H^+^]_i_ are given, which take into account the different pH baseline (see also: [34]). The amplitude and the rate of change of the measured [H^+^]_i_ were analyzed.

### Voltage clamp recording

A borosilicate glass capillary, 1.5 mm in diameter, was pulled to a micropipette and backfilled with 3 M KCl. The resistance of the electrodes measured in oocyte saline was around 1 MΩ. For voltage-clamp, both electrodes were connected to the head-stages of an Axoclamp 2B amplifier (Axon Instruments, USA). The experimental bath was grounded with a chlorided silver wire coated by agar dissolved in oocyte saline. Oocytes were clamped to a holding potential of −40 mV.

### Fluorescent staining of CA in oocytes

Oocytes, either injected with cRNA for CAI, II and III as well as the mutants CAII-V143Y, -Y7F and -H64A, and native control oocytes, were fixed in 4% paraformaldehyde in phosphate-buffered saline (PBS). Oocytes were treated with 100% methanol and permeabilized with 0.1% Triton X-100. Unspecific binding sites were blocked with 3% bovine serum albumin (BSA; Sigma-Aldrich) and 1% normal goat serum or 1% donkey serum, depending on the secondary antibody. The cells were incubated in 1% BSA with 0.01% Triton X-100 containing the primary antibodies against CAI (goat anti-human CAI, Lifespan Biosciences), CAII (rabbit anti-CAII, Chemicon) and CAIII (goat anti-human CAIII, Santa Cruz Biotechnology, Inc.) overnight at 4°C. Oocytes were then incubated with the secondary antibody (Alexa Fluor 488 goat anti-rabbit IgG or donkey anti-goat IgG, Invitrogen, Darmstadt, Germany). The stained oocytes were analyzed with a laser scanning microscope (LSM 510, Carl Zeiss GmbH, Oberkochen, Germany), using whole oocytes, through which cross sectional optical planes were laid.

Adobe Photoshop CS3 (Adobe Systems Inc., USA) was used to process the images; all images of oocytes expressing the same CA-isoform and corresponding controls (native oocytes) were treated identically.

### Quantification of CA expression by Western blot analysis

20 oocytes, expressing CAII, -H64A, -Y7F or -V143Y alone or coexpressing NBCe1+CAII, as well as native oocytes (control), were lysed in 200 µl 2% SDS (MP Biomedicals, Illkirch, France) with protease inhibitors (Complete Mini EDTA-free, Roche Diagnostics GmbH, Mannheim, Germany) by sonication. After determination of total protein concentration (BCA™ Protein Assay Kits; Thermo Scientific, Rockford, USA), 12 or 15 µg total protein of oocytes were loaded on a 4–12% NuPage^R^ Novex^R^ Bis-Tris Mini Gel (Invitrogen, Carlsbad, USA) under reducing conditions. As protein standard, 5 µl Novex^R^ Sharp Pre-stained Protein Standard (Invitrogen) was used. Gel electrophoresis was performed with NuPage^R^ MOPS SDS Running Buffer (Invitrogen) in a XCell Sure Lock™ Electrophoresis Cell (Invitrogen). Proteins were transferred on a nitrocellulose membrane (0.45 µM; Invitrogen) by Western blotting. The membrane was blocked for 1 hour in 50 mM Tris-HCl, pH 7.5, 150 mM NaCl, 0.2% Tween 20 and 5% skimmed dry milk (TBST+L) before it was incubated with the primary antibody against CAII (1∶500; rabbit anti-CAII, Chemicon) overnight at 4°C. After washing in TBST, the incubation of the secondary antibody (1∶4000; Goat anti-rabbit IgG-HRP, Santa Cruz) in TBST+L for 1 hour at room temperature was performed. LumiLight Western Blotting Substrate (Roche) was added and the CAII detected by Lumi-Imager (VersaDoc Imaging System Model 3000; Biorad). As loading control, β-tubulin was labeled with anti-β-tubulin mouse monoclonal antibody (1∶2000; Sigma Aldrich). The PC-program Quantity One (Biorad) was used for quantification analysis. After normalization of the loading control, corrected values of CAII-stainings were normalized to oocytes expressing wild-type CAII. Corel Draw X3 (Corel Corp.) was used to produce the final figures.

### Determination of CA activity by mass spectrometry

Activity of CAI, II and III, as well as the mutant CAII-V143Y, was determined by monitoring the ^18^O depletion of doubly labeled ^13^C^18^O_2_ through several hydration and dehydration steps of CO_2_ and HCO_3_^−^ at 25°C [35], [36]. The reaction sequence of ^18^O loss from ^13^C^18^O^18^O (m/z = 49) over the intermediate product ^13^C^18^O^16^O (m/z = 47) and the end product ^13^C^16^O^16^O (m/z = 45) was monitored continuously with a quadrupole mass spectrometer (MSD 5970; Hewlett Packard, Waldbronn, Germany). The relative ^18^O enrichment was calculated from the measured 45, 47, and 49 abundance as a function of time according to: log enrichment = log [49×100/(49+47+45)]. For the calculation of the CA activity of the sample, the rate of ^18^O degradation was obtained from the linear slope of the log enrichment over the time, using the spreadsheet analyzing software Origin 7.0 (OriginLab Corp., Northampten, MA). The rate was compared with the corresponding rate of the non-catalyzed reaction before application of oocytes or CA-protein into the cuvette. Enzyme activity in units (U) was calculated from these two values as defined by Badger & Price [37]. From this definition, one unit corresponds to 100% stimulation of the non-catalyzed ^18^O depletion of doubly labeled ^13^C^18^O_2_. For the experiments, the cuvette was filled with 8 ml of oocyte saline with a pH of 7.35 according to the mean pH_i_ of oocytes (data not shown), 8 µl of doubly labeled substrate was added. Experiments were carried out at 25°C. To determine the catalytic activity, for each sample, 20 intact, CA-expressing oocytes were pipetted into the cuvette. Inside the cuvette the oocytes were rapidly lysed by stirring, allowing measurement of CA activity. For calibration of CA activity, 0.25, 0.5, 1 and 2 µg of CAI- and CAII-protein were directly added to the cuvette and compared to the activity of oocytes.

### Calculation and statistics

Statistical values are presented as means ± one standard error of the mean (S.E.M.). For calculation of significance in differences, ANOVA followed by Fishers LSD test was used (OriginPro 8; OriginLab Corp.). In the figures shown, a significance level of p≤0.05 is marked with *, p≤0.01 with ** and p≤0.001 with ***.

## Results

### Expression of CA-isoforms and mutants of CAII

**Figure 1:**
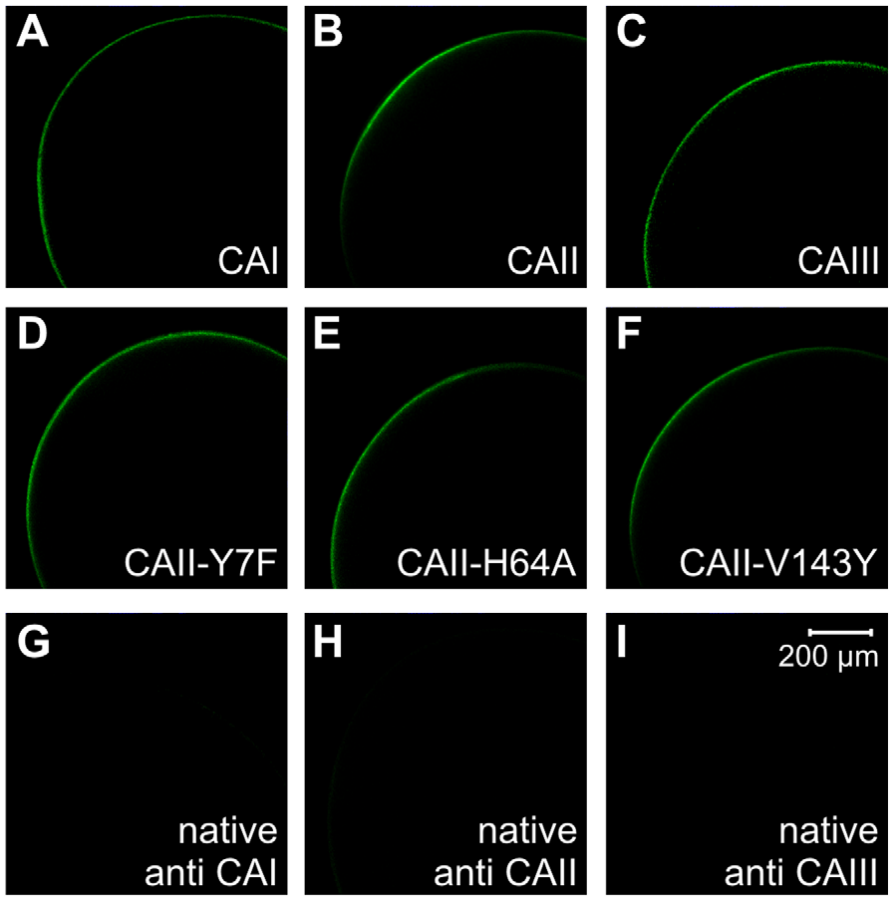
Fluorescent staining of CA isoforms and mutants. Optical slices of oocytes, labeled via primary antibodies and Alexa Fluor 488-linked secondary antibodies against CAI (A), CAII (B), CAIII (C), CAII-Y7F (D), -H64A (E) and -V143Y-expressing oocytes (F). As control, a staining of native, uninjected oocytes or oocytes injected with H_2_O with primary antibodies against CAI (G), CAII (H) and CAIII (I) as well as corresponding secondary antibodies, respectively, was performed.

CA-expression was demonstrated by confocal images, taken from CAI, II and III-expressing oocytes, as well as CAII-Y7F, -H64A and -V143Y-expressing, and native oocytes, stained with antibodies against CAI, CAII and CAIII, respectively. All CA isoforms and mutants could be detected in the oocyte. In optical slices the signal was restricted to the plasma membrane (Fig. 1). Since using optical slices of whole oocytes results in limitations of antibody diffusion and detection of fluorescence inside the oocytes, the stainings are controls of expression of CA-isoforms in the oocytes without determination of the exact cellular location.

### Catalytic activity of CAI, II and III

**Figure 2:**
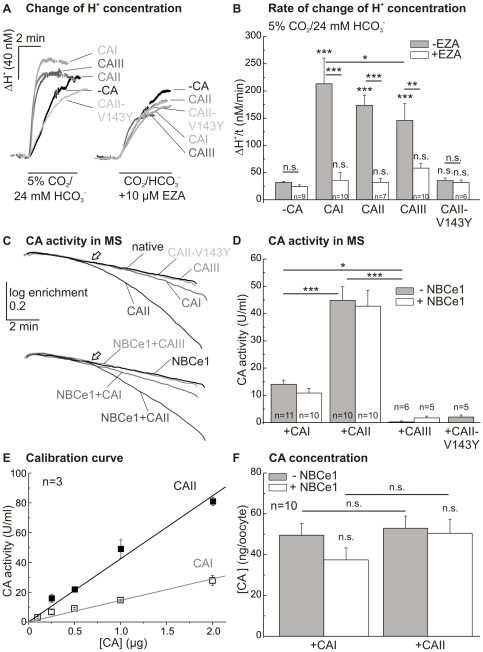
Activity of CAI, II and III. Original recordings of changes of intracellular proton concentration (ΔH^+^; A) and statistical analysis of the rates of rise of proton concentration (ΔH^+^/t; B) after application of 5% CO_2_/24 mM HCO_3_^−^-buffered solution before or during application of EZA (10 µM). The asterisks above the bars correspond to the control cells without CA (−CA) before (−EZA) or during application of EZA (+EZA). (C) Original recordings of the log enrichment of 20 oocytes expressing CAI, CAII and CAIII, respectively, either alone or together with the NBCe1, or 20 CAII-V143Y, native or just NBCe1-expressing oocytes as measured by mass spectrometry (MS). The arrows indicate the application of 20 intact oocytes, which were rapidly lysed inside the cuvette by stirring. (D) Statistical analysis of CA activity (U/ml) after subtraction of either native (4 U/ml; 20 oocytes, n = 3) or NBC-expressing oocytes (5 U/ml; 20 oocytes, n = 3), as obtained by mass spectrometry. Oocytes expressing the catalytically inactive mutant CAII-V143Y were used as control. Calibration curve of different CAI- and CAII-protein concentrations (E) to determine the amount of CA-protein expressed in oocytes (F). The asterisks above the bars for NBCe1-coexpressing oocytes (+NBCe1, 13.8 ng NBCe1-RNA) correspond to those without NBCe1-coexpression (−NBCe1). A significance level of p≤0.05 is marked with *, p≤0.01 with ** and p≤0.001 with ***.

We expressed CAI, CAII and CAIII, as well as the mutant CAII-V143Y, in oocytes, to check for differences in catalytic activity between the different CA-isoforms. Catalytic activity was measured by pH-sensitive microelectrodes during application of 5% CO_2_/24 mM HCO_3_^−^-buffered solution before and after addition of the CA-inhibitor ethoxyzolamide (6-ethoxy-2-benzothiazolesulfonamide; EZA; 10 µM; Fig. 2 A). The rate of rise of proton concentration was enhanced 4.5- to 6.6-fold by CAI, II and III, with only slight changes between the different isoforms (Fig. 2 B). Expression of the different CA-isoforms had no effect on the intrinsic buffer capacity of the oocytes (native: 16.1±1.4 mM; CAI: 13.6±1.8 mM; CAII: 14.0±1.2 mM; CAIII: 15.4±1.8 mM; p = 0.68). Catalytic activity of all three isoforms was inhibited by EZA, but while CAI and CAII were blocked completely, CAIII showed only partial inhibition (Fig. 2 B). Expression of CA isoforms led to no significant difference in the absolute change of the proton concentration during application of CO_2_/HCO_3_^−^ (native: 97±6 nM; CAI: 133±25 nM; CAII: 107±7 nM; CAIII: 107±15 nM; and after addition of EZA: 80±10 nM, 90±31 nM, 76±12 nM and 72±9 nM, respectively; p = 0.26). The catalytically inactive mutant CAII-V143Y did not show an increase of rate of rise of proton concentration during application of CO_2_/HCO_3_^−^-buffered solution as compared to native oocytes, and there was no reduction of the rate of rise of proton concentration in the presence of EZA with this mutant (Fig. 2 B).

Another, independent, method for the determination of CA activity is the CA-catalyzed degradation of ^18^O-labeled HCO_3_^−^, measured by mass spectrometry. Fig. 2 C shows ^18^O-depletion after addition of 20 oocytes expressing either CA alone or coexpressing CA with NBCe1. Catalytic activity of CAII- (p≤0.001) and CAI-expressing oocytes (p≤0.05 or p≤0.01), either with or without coexpression of NBCe1, was increased as compared to native or NBCe1-expressing control cells. CAIII-expressing oocytes showed no measurable catalytic activity in mass spectrometry, neither if expressed alone nor when coexpressed with NBCe1. CAII-V143Y-expressing oocytes also showed no measurable catalytic activity (see also: [12]). After subtraction of background activity of the control oocytes (native: 4 U/ml and NBCe1-expressing: 5 U/ml; per 20 oocytes, n = 3), CAII-expressing oocytes showed the highest catalytic activity, and coexpression with NBCe1 did not significantly alter this value (Fig. 2 D), while CAI-expressing oocytes showed only about 30% of the activity of CAII-expressing oocytes (p≤0.001).

By means of mass spectrometric analysis, the amount of CAI- and CAII-protein expressed in the oocytes was determined. For this, catalytic activity of different concentrations of CAI- or CAII-protein, directly added to the cuvette, was measured to create a calibration curve (Fig. 2 E). This curve was used to determine the concentration of active CAI and CAII expressed in oocytes after injection of 11.5 ng cRNA for CAI and CAII, respectively (Fig. 2 F). The calculations revealed no difference in the amount of expressed protein between CAI and CAII. Furthermore, coexpression with NBCe1 did not significantly affect the amount of expressed CAI or CAII. Due to the low catalytic activity of CAIII in mass spectrometry, the protein amount of this isoform could not be calculated. However, in Western blot analysis, a concentration of 65±14 ng/oocyte was recently reported [26].

### Effect of CAI and III expression on NBCe1-transport activity

**Figure 3:**
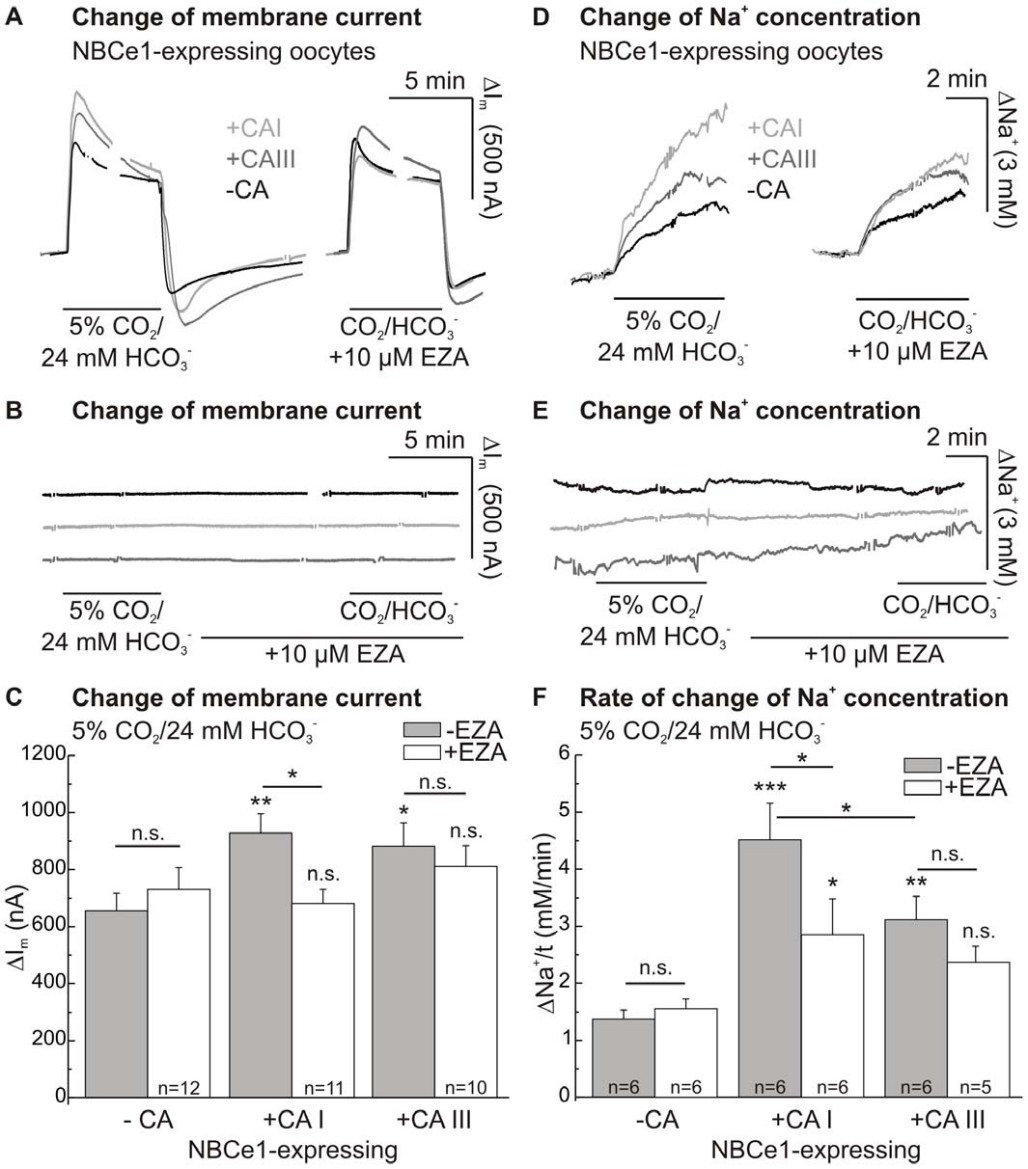
Effect of CAI or CAIII on NBCe1 transport activity. Original recordings (A) and statistics of the changes in membrane current (ΔI_m_; C) of NBCe1- and NBCe1+CAI- or NBCe1+CAIII-expressing oocytes during application of 5% CO_2_/24 mM HCO_3_^−^-buffered solution in the absence and presence of EZA (10 µM). Original recordings of ΔI_m_ of CA-expressing control cells without coexpression of NBCe1 (B). By the use of Na^+^-selective microelectrodes the rates of rise of intracellular sodium concentration (ΔNa^+^/t; D, F) were obtained. Original recordings of ΔNa^+^/t of control cells without coexpression of NBCe1 (E). The asterisks above the bars correspond to the control cells without CA expression (−CA) before (−EZA) or during application of EZA (+EZA).

Transport activity of NBCe1, expressed in *Xenopus* oocytes, was determined by measuring the membrane current and the intracellular sodium concentration of oocytes, voltage-clamped to a holding potential of −40 mV. NBCe1 was activated by changing from a HEPES- to a 5% CO_2_/24 mM HCO_3_^−^-buffered solution (pH 7.4). This induced an outwardly directed membrane current and a rise in intracellular sodium concentration in NBCe1-expressing oocytes, indicating inwardly directed, electrogenic, transport of Na^+^ and HCO_3_^−^ via NBCe1 (Fig. 3 A, D). There was no significant change in current (2–9 nA; n = 8–9) and intracellular sodium concentration (0.02–0.07 mM/min; n = 4) in oocytes without NBCe1 when introducing CO_2_/HCO_3_^−^ (Fig. 3 B, E).

CAI-coexpressing oocytes showed a CO_2_/HCO_3_^−^-induced membrane current of 928±68 nA (n = 11), CAIII-coexpressing cells of 883±81 nA (n = 10), while oocytes just expressing NBCe1 showed a membrane current of only 656±60 nA (n = 12; Fig. 3 C). In the presence of the CA-inhibitor EZA (10 µM), the application of CO_2_/HCO_3_^−^-buffered solution led to a reduced change of membrane current in CAI-coexpressing oocytes. On the other hand, with a change of membrane current of 827±57 nA (n = 9), coexpression of NBCe1 with the catalytically inactive mutant CAII-V143Y led to no significant augmentation of the membrane current during application of CO_2_/HCO_3_^−^-buffered solution, compared to NBCe1-expressing control oocytes with 733±50 nA (n = 9). Furthermore, application of EZA did not induce any significant change of CO_2_/HCO_3_^−^-induced membrane current, which was 814±59 nA in CAII-V143Y-coexpressing oocytes as compared to 785±43 nA in oocytes expressing NBCe1 alone (see also: [12]).

The rate of rise of intracellular sodium concentration was also increased during application of CO_2_/HCO_3_^−^-buffered solution in CAI- (3.3-fold) and CAIII-coexpressing oocytes (2.3-fold) as compared to NBCe1-expressing oocytes without CA (Fig. 3 F). The increase of the NBCe1-transport activity between the two CA-isoforms was not significantly different in membrane current, but was different in rate of rise of intracellular sodium concentration (p≤0.05). Like the increase of membrane current, the increase of the rate of rise of the intracellular Na^+^-concentration was reduced by EZA in the case of CAI. Oocytes coexpressing NBCe1 showed no significantly lower rate of rise of the sodium concentration or reduced membrane current associated with the less EZA-sensitive CAIII in the presence of the CA-inhibitor.

### Effect of injection of different concentrations of CAI-protein on catalytic activity and NBCe1 transport activity

**Figure 4:**
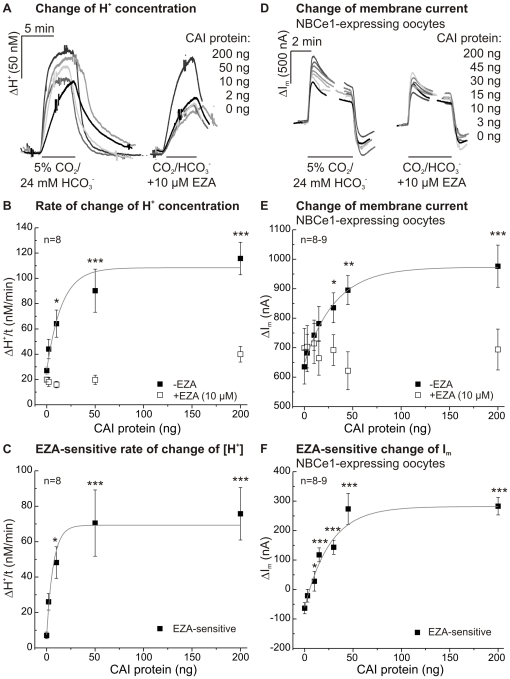
Effect of CAI-protein injection (0–200 ng) on the catalytic activity and NBCe1 transport activity. Original recordings of the changes of intracellular proton concentration of CAI-injected oocytes (A) and of membrane current of CAI-injected, NBCe1-expressing oocytes (D) after application of 5% CO_2_/24 mM HCO_3_^−^-buffered solution in the absence and presence of EZA (10 µM). Rates of rise of proton concentration (B) and changes of membrane current (E) before (filled squares) or during (open squares) EZA application in CO_2_/HCO_3_^−^-buffered solution. EZA-sensitive rates of rise of proton concentration (C) and changes of membrane current were plotted (F). The asterisks correspond to the control cells without CA-injection (0 ng CAI).

The effect of CAI on NBCe1, which could be suppressed by EZA, was studied in more detail by injecting different concentrations of CAI-protein (0–200 ng) directly into NBCe1-expressing oocytes 12–24 hours before the measurements were carried out. Both, catalytic activity and NBCe1 transport activity were enhanced in these oocytes with increasing concentration of CAI-protein (Fig. 4 A, D).

The rate of rise of proton concentration during application of 5% CO_2_/24 mM HCO_3_^−^-buffered solution in oocytes, indicative for CAI catalytic activity, is illustrated in Figure 4 B for oocytes without NBCe1 before (filled squares) and during application of EZA (open squares). Half-maximal activity was determined at a concentration of 5–10 ng CAI, maximal activity was obtained at about 50 ng of protein. Even after injection of 10 ng CAI-protein, a significant EZA-sensitive rate of rise of proton concentration could be detected, as revealed in a plot, where the EZA-sensitive rate of H^+^ rise was isolated by subtraction of the values in the absence and presence of EZA (Fig. 4 C).

The effect of CAI on the transport activity of NBCe1 was determined by evaluating the membrane current of NBCe1-expressing oocytes (Fig. 4 D), before (filled squares) and during application of EZA (open squares, Figure 4 E). Subtraction of the two curves resulted in the EZA-sensitive change in membrane current, indicating the NBCe1 transport activity as augmented by catalytic activity of CAI (Fig. 4 F). Half-maximal effect on the NBCe1 activity was obtained with an injection of about 15 ng CAI-protein.

### Activity and effect of CAII-H64A and -Y7F mutants on NBCe1 transport activity

**Figure 5:**
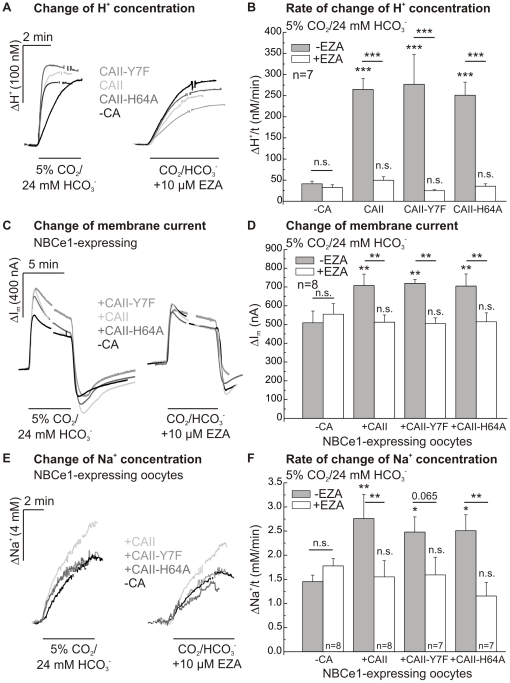
Activity and effect of CAII mutants on NBCe1 transport activity. Changes of the intracellular proton concentration as recorded in native, CAII-, CAII-H64A- and CAII-Y7F-expressing oocytes (A) to determine rates of rise of the proton concentration (B). Original recordings of the changes in membrane current (C) and intracellular sodium concentration (E) in NBCe1 and CAII-, CAII-H64A- or CAII-Y7F- coexpressing oocytes after application of 5% CO_2_/24 mM HCO_3_^−^-buffered solution before or during application of EZA (10 µM). Statistical analysis of membrane current (D) and rates of rise of intracellular sodium concentration (F) revealed an increase in NBCe1 transport activity after coexpression of either CAII or one of the two mutants. The asterisks above the bars correspond to the control cells without CA (−CA) before (−EZA) or during EZA (+EZA) application.

The enzymatic activity of the two CAII mutants, CAII-H64A and -Y7F, was determined by pH-sensitive microelectrodes during application of CO_2_/HCO_3_^−^-buffered solution before and during application of EZA (10 µM). The rate of rise of proton concentration was 6–7-fold increased by CAII, CAII-Y7F and -H64A (p≤0.001; Fig. 5 A, B). In the presence of EZA, the activity of CAII and both mutants was completely blocked. The absolute changes of proton concentration did not differ between the three CA-expressing cell types and native oocytes (native: 94±17 nM; CAII: 121±6 nM; CAII-Y7F: 108±8 nM; CAII-H64A: 113±11 nM; and after addition of EZA: 80±22 nM, 104±11 nM, 75±5 nM and 90±11 nM, respectively; p = 0.1).

A potential role of the CA's intramolecular proton shuttle for the CAII-mediated augmentation of NBCe1 transport activity was studied using these CAII mutants. The transport activity of NBCe1, which was coexpressed with either mutant CAII-H64A and -Y7F, in *Xenopus* oocytes, was determined by measuring the membrane current and intracellular sodium concentration of oocytes voltage-clamped to a holding potential of −40 mV during application of a 5% CO_2_/24 mM HCO_3_^−^-buffered solution.

Coexpression of NBCe1 with CAII-Y7F and -H64A induced a significant increase in membrane current, as compared to oocytes expressing NBCe1 alone (p≤0.01; Fig. 5 C, D). No significant difference in the CA-induced augmentation of NBCe1 transport activity was observed between CAII and the two CAII mutants. EZA, which blocked the CA activity of both mutants and CAII, also reversed the increase in NBCe1 transport current.

CAII and both CAII mutants also induced a significant increase in the rate of NBCe1-mediated change in intracellular sodium concentration during application of CO_2_/HCO_3_^−^-buffered solution (Fig. 5. E, F). Again, there was no difference in the augmentation of NBCe1 transport activity between CAII and the two CAII mutants. Similar to the membrane current, there was a complete blockade of the CA-mediated increase in the rate of sodium rise by EZA in CAII and CAII-H64A-coexpressing cells (p≤0.01), whereas the blockade was not significant in CAII-Y7F-coexpressing oocytes (p = 0.065). There was no change in membrane current (−2 nA–14 nA; n = 7–9) and intracellular sodium concentration (0.0–0.2 mM/min; n = 3–5) in control oocytes without NBCe1.

### Effect of injected CAII-protein on NBCe1 transport activity at different bicarbonate concentrations and constant CO_2_

**Figure 6:**
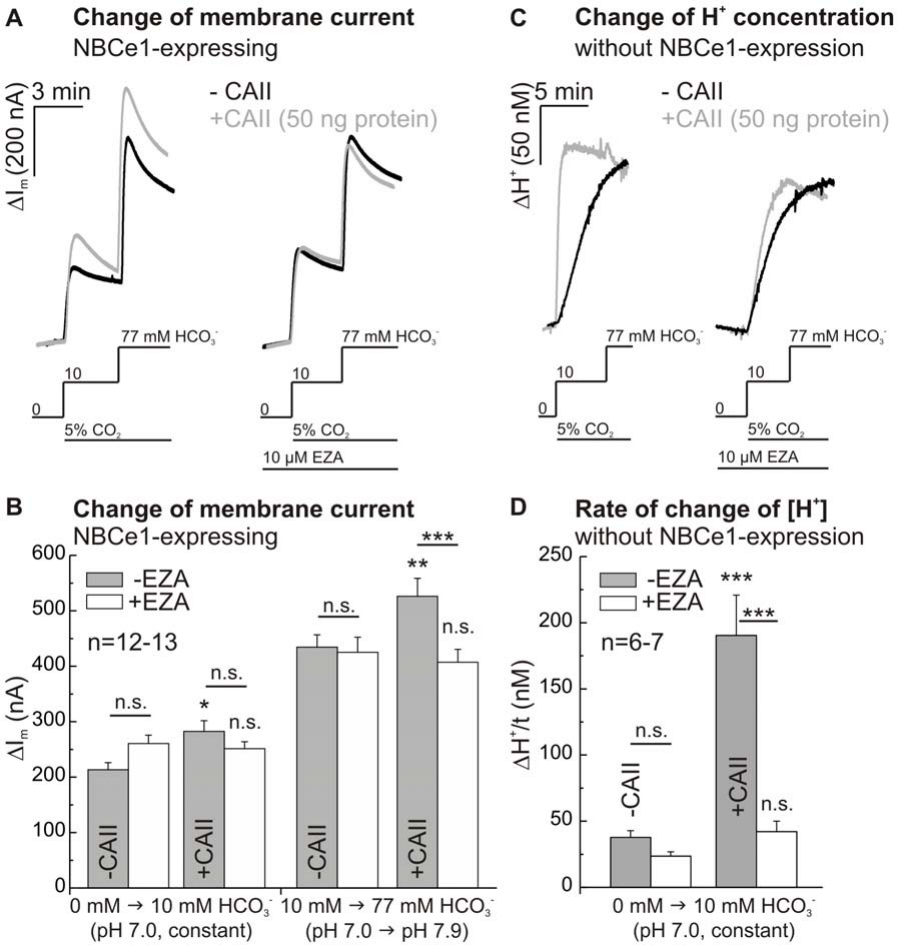
Effect of injected CAII-protein on NBCe1 transport activity at different bicarbonate concentrations and constant CO_2_. Original recordings of membrane current (A) in NBCe1-expressing oocytes with or without injection of 50 ng CAII-protein after changing from a HEPES-buffered, bicarbonate-free saline (pH 7.0) to a 5% CO_2_/10 mM HCO_3_^−^-buffered saline (pH 7.0) as well as after increasing the HCO_3_^−^ concentration from 10 mM (pH 7.0) to 77 mM HCO_3_^−^ (pH 7.9) in a 5% CO_2_-equilibrated saline, before or during application of EZA (10 µM). Statistical analysis of membrane current (B) revealed an increase in NBCe1 transport activity after injection of CAII-protein in both salines. Original recordings of the change of intracellular proton concentration (C) and statistical analysis of rate of rise of proton concentration (D) of native oocytes and oocytes injected with CAII-protein. The asterisks above the bars correspond to the control cells without CA (−CA) before (−EZA) or during EZA (+EZA) application.

To exclude effects of a CO_2_-mediated acidification during the initial phase of application of 5% CO_2_/24 mM HCO_3_^−^-buffered solution, we changed the bicarbonate concentration from 10 mM to 77 mM in the constant presence of 5% CO_2_. The transport activity of NBCe1-expressing oocytes injected with 50 ng CAII-protein, both after changing from a HEPES-buffered, bicarbonate-free saline (pH 7.0) to a 5% CO_2_/10 mM HCO_3_^−^-buffered saline, as well as after increasing the HCO_3_^−^ concentration from 10 mM (pH 7.0) to 77 mM HCO_3_^−^ (pH 7.9) in a 5% CO_2_-equilibrated saline, was increased as compared to NBCe1-expressing oocytes without injection of CA (Fig. 6 A, B). The enhancement of NBCe1 transport activity after increasing the HCO_3_^−^ concentration from 10 mM to 77 mM HCO_3_^−^ in a 5% CO_2_-equilibrated saline could be reversed by EZA (10 µM). There was no change in membrane current in control oocytes without NBCe1 (n = 4). Catalytic activity of CAII-protein was confirmed by a 5-fold increase in the rate of rise of proton concentration, induced by application of 5% CO_2_/10 mM HCO_3_^−^, in oocytes injected with 50 ng CAII-protein, as compared to water-injected native oocytes (Fig. 6 C, D). These results indicate that the enhancement of NBCe1 transport activity by CA activity is independent of a change in CO_2_ concentration.

### Quantification of CAII-expression

**Figure 7:**
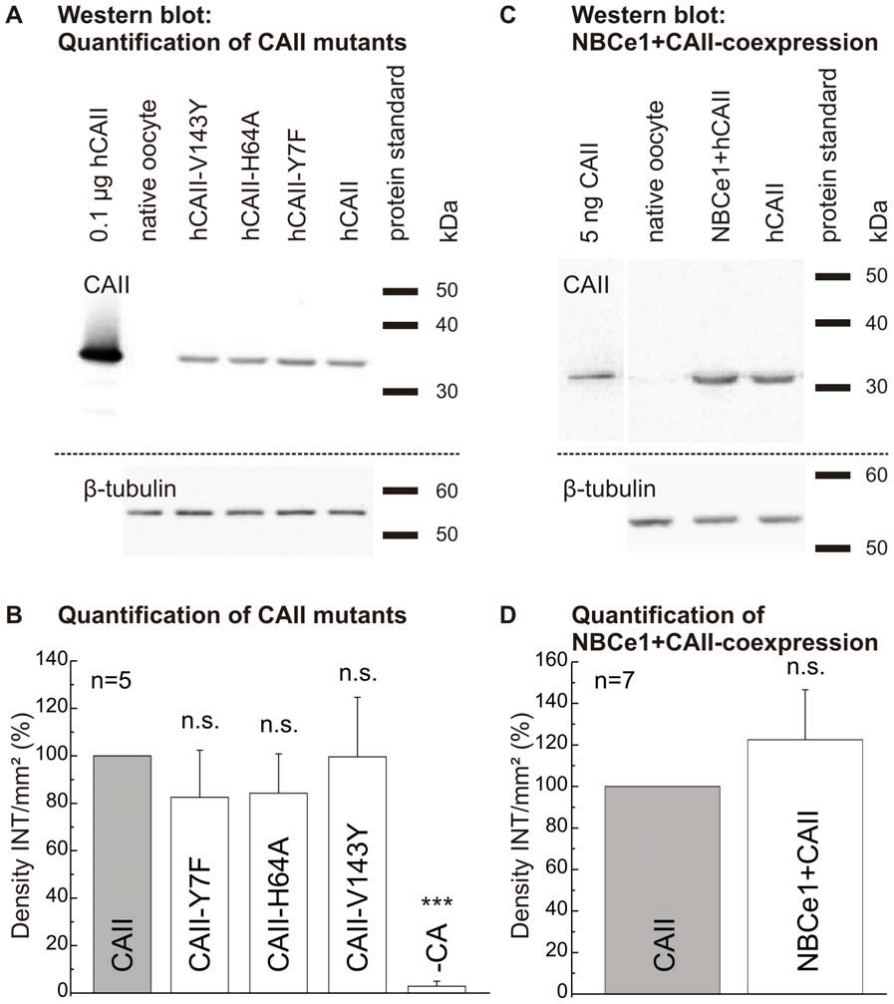
Quantification of CAII mutants. Western blot of the different CAII mutants (CAII-Y7F, CAII-H64A and CAII-V143Y) as well as wild-type CAII (A; 12 µg total protein/lane), β-tubulin was used as loading control, and quantification of the expression of the CAII mutants in oocytes, as compared to wild-type by determination of the density of intensity (B; Density INT/mm^2^). Western blot of CAII-expressing and NBCe1+CAII-coexpressing oocytes, with β-tubulin used as loading control (C; 15 µg total protein/lane) and quantification of effect of NBCe1 coexpression on CAII expression rate (D).

A quantification of the expression of CAII and the different CAII mutants by Western blot was performed to reveal a potential influence of NBCe1 expression on the amount of expressed CA. The Western blots showed a band corresponding to CAII at 35 kDa. Native oocytes did not show any bands in the Western blot. Statistical analysis indicated no significant difference between the CAII mutants H64A, Y7F and V143Y in comparison to CAII-expressing oocytes normalized to 100% density INT/mm^2^, suggesting similar CA expression of wild-type and CAII mutants (Fig. 7 A, B).

Figure 7 C shows a Western blot for the quantification of CAII in NBCe1/CAII-coexpressing oocytes with a band corresponding to CAII at about 30–35 kDa. In comparison to CAII-expressing oocytes normalized to 100% density of intensity/mm^2^, NBCe1+CAII-coexpressing cells showed no significant change in the expression of CAII (n = 7; Fig. 7 D). The loading control β-tubulin was used as expression level standard in these calculations, as described in the Methods section.

## Discussion

### Effect of CAI, CAII and CAIII on NBCe1 transport activity

We have recently shown that NBCe1 transport activity is enhanced by coexpression of CAII or after injection of CAII-protein into *Xenopus* oocytes [12], and that catalytic activity of CAII is important for this interaction. In contrast, other groups could not find such an interaction between NBCe1 and CAII by examining the change of membrane conductance [13] or determining change of membrane current [14]. We have now investigated the effect of two other intracellular isoforms of CA, CAI and CAIII, on the transport activity of NBCe1 after expression of the proteins in *Xenopus* oocytes. By recording the membrane current and the intracellular sodium concentration, both robust parameters of NBCe1 transport activity, during application of CO_2_/HCO_3_^−^-buffered solution, we were able to show that NBCe1 transport activity is enhanced by each of these isoforms to a similar extent (Fig. 3). The effect of CAI could be reversed, by blocking the catalytic activity with the CA-inhibitor EZA. CAIII exhibits less sensitivity against sulfonamides due to a reduced accessibility of its active site [38], and hence its effect on NBCe1 activity was not significantly reduced by EZA. In addition, we were able to show that the enhancement of NBCe1 by CAII occurs also by changing the HCO_3_^−^ concentration under constant CO_2_-equilibration. Our results show that there is a functional interaction between NBCe1 and different intracellular CA-isoforms, which results in an increased transport activity by NBCe1 allowing more efficient acid-/base-regulation.

### CA activity in vitro

It was reported that the catalytic activity of CAI is about 15% [18], [19] and that of CAIII about 0.3% of the activity of CAII [15]–[17]. This difference in catalytic activity was attributed to the difference in the amino acids lining the active site of the respective enzymes. The catalytic site of CAII has, besides the three zinc ligands, only one further histidine (His64), whereas the catalytic site of CAI exhibits three additional histidines [39], [18]. A further major difference is a histidine-rich cluster of CAII, which reaches from the middle of the active site to the surface of the protein and acts presumably as a proton shuttle [40]. CAIII does not possess a histidine on position 64, but instead a lysine (Lys64), which is less effective as a proton shuttle [38], [41], [42]. Furthermore, in position 198, CAIII has a phenylalanine, which has a very bulky side chain and seems to reduce CAIII activity even more [43]. Whereas the physiological function of CAII is well understood, the functional roles of CAI and CAIII are much less clear. CAIII is expressed in high concentrations in the red skeletal muscle, while CAI is expressed primarily in red cells, gastrointestinal epithelia or vascular epithelium (for review see: [44]).

We also checked the catalytic activity of the three isoforms in mass spectrometry under *in vitro* conditions. We were able to confirm the results of the *in vitro* measurements of other groups [15], [19]. We found for CAI-expressing oocytes about 30% activity, whereas CAIII-expressing oocytes, as well as oocytes expressing the catalytic inactive mutant CAII-V143Y, did not show any measurable catalytic activity. The expression of CAI and CAII in oocytes was equal (about 50 ng/oocyte), and no effect on the expression level of CAI or CAII was observed when coexpressed with NBCe1. Protein expression of CAIII on the other hand could not be determined by the use of mass spectrometry because of the low catalytic activity in the *in vitro* measurement. However, we have recently shown by Western blot analysis a CAIII concentration of 65±14 ng/oocyte [26].

### CA activity in intact oocytes

Both CAI and CAIII showed significant catalytic activity in intact oocytes, as determined by the rate of acidification during application of CO_2_/HCO_3_^−^, confirming our previous studies on CAI [45] and CAIII [26]. The intrinsic buffer capacity was not altered by the expression of either CA-isoform. However, oocytes expressing the catalytically inactive mutant CAII-V143Y did not show any activity, as expected, similar to that of native oocytes.

It appears that the activity of CAI and CAIII has been restored or activated by a yet unknown factor in the cytosol, while CAII activity might be reduced. Scozzafava and Supuran [46] have shown an increase of catalytic activity of CA by micromolar concentrations of histamine or ‘new activators’, such as e.g. carnosine and imidazole derivates, in human erythrocytes. Histidine-containing dipeptides like carnosine, homocarnosine and anserine have also been detected as intracellular mobile H^+^-buffers in cardiac and skeletal muscle [47]–[49]. Jonsson et al. [50] have shown an increase of CAII activity by several buffers, e.g. 1,2-dimethylimidazole or 2,2-diethylmalonate. The activity of CAIII was also shown to be increased by small mobile buffers, especially imidazole, in membrane-inlet mass spectrometry and stopped-flow spectrophotometry [30], [51].

The restoration or increase of CA activity in intact oocytes might explain that all three catalytically active isoforms enhanced transport activity of NBCe1. It can be speculated that if catalytic activity of CAs could be rescued by substances in the cytosol of oocytes, catalytic activity might be suppressed due to the absence of these yet unknown factors in *in vitro* measurements due to the high dilution of the cytosol with HEPES-buffered solution, as e.g. in mass spectrometry. Recently, the membrane conductance associated with the glutamine transporter SNAT3 (SLC38A3) was shown to be suppressed not only by CAII activity [52], but also by CAI and CAIII [53].

### Functional interaction of CA and NBCe1

In addition to the finding of this study that CAI and CAIII have an enhancing effect on transport activity of NBCe1, we have also investigated the effect of injection of different concentrations of CAI on catalytic activity and NBCe1 transport activity. The effect of CAI on NBCe1 transport activity increased with the concentration of CAI and hence in parallel with the catalytic activity of CA. Even an injection of 10 ng CAI led to a detectable catalytic CA activity, and injection of 10 ng CAI resulted in a significant increase of EZA-sensitive NBCe1 activity. Maximal CA activity and enhancement of NBCe1 transport activity was obtained after injection of 45–50 ng CAI. The values for CAI activity fit well to previous measurements, which gave an EC_50_ of 11.0±1.6 ng CAI/oocyte and a near maximal rate of acidification at ∼50 ng CAI [45]. This means that oocytes expressing or coexpressing CAI with about 50 ng per oocyte, as used in this study, show near maximal catalytic activity as well as near maximal effect on NBCe1 transport activity in oocytes, similar as previously shown for CAII in oocytes [12].

We also investigated a potential role of the intramolecular proton shuttle in CAII on NBCe1 transport activity by coexpression of the mutants CAII-H64A and -Y7F together with the bicarbonate transporter. This intramolecular proton shuttle was shown to be essential for the CAII-mediated increase in transport activity of the monocarboxylate transporters (MCT) 1 and 4 [26]. When coexpressed with NBCe1, both mutants showed the same effect on NBCe1 transport activity as described for the wild-type CAII, as demonstrated by a similar increase of membrane current and rate of rise of intracellular sodium concentration during application of CO_2_/HCO_3_^−^-buffered solution. It may be speculated, that the proton transfer of CA can be rescued by substances in the cytosol of the oocytes so that catalytic activity is restored. The CAII-H64A mutant, which was shown to exhibit less than 10% of wild type enzyme activity in *in vitro* measurements [20], [21], had indeed similar catalytic activity as CAII-WT and CAII-Y7F, as determined from the rate of rise of intracellular proton concentration, in intact oocytes. This increase in CAII-H64A activity is not attributable to an altered expression level of wild-type CAII and CAII mutants, as shown by a quantitative Western blot analysis of CAII expression (Fig. 7). We were also able to show by quantitative Western blot analysis that there is no effect on the expression level of CAII when coexpressing NBCe1 with CA. In contrast to another report [14], we were able to show that there is very little endogenous CAII present in our *Xenopus* oocytes. Equal expression of NBCe1 was confirmed by a comparison of NBCe1 transport activity of NBCe1 alone and when coexpressed with CA in the presence of the CA-inhibitor EZA (Fig. 5 D, F).

**Figure 8:**
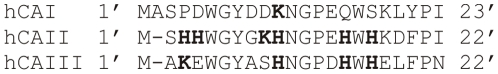
Potential binding motif (bold amino acids) of hCAII (Swiss-Prot.: P00918) against NBCe1 is incompletely conserved in N-terminus of intracellular hCAI (Swiss-Prot.: P00915) or hCAIII (Swiss-Prot.: P07451).

By the use of the three different intracellular CA-isoforms, we were also able to gain insight into the type of interaction between CA and NBCe1. For anion exchanger 1 (AE1), which is, like NBCe1, a member of the SLC4-family of bicarbonate transporters, a direct binding between an acidic motif in intracellular C-term of AE1 (D^887^ADD) and a basic binding motif in N-term of CAII was suggested [27], [54]. Furthermore, Pushkin et al. [11] have shown a direct binding between an acidic motif in the intracellular C-terminal of NBCe1 (L^958^DDV and D^986^NDD) and immobilized CAII. Another report, however, could neither detect a binding of CAII to the immobilized pure NBCe1- or AE1-Ct peptides, nor detect possible rapid interactions between CAII and the pure peptides in surface plasmon resonance spectroscopy [55]. Even though CAI lacks the histidine cluster, which is suggested to bind to the C-terminal of AE1-3 and NBCe1, and although CAIII contains only parts of this cluster (Fig. 8), both CA-isoforms showed a similar effect on NBCe1 transport activity as CAII does. Furthermore, we have shown previously that mutation of this histidine-rich cluster (H3P, H4Q, K9A, H10K, H15Q, H17S) does not impair the CAII-induced augmentation of NBCe1-transport activity [12], while the interaction between MCT1 and CAII was abolished by this mutation [45]. Therefore, the CAII-mediated augmentation of NBCe1 transport activity may not require the basic binding motif within the N-terminal of the enzyme.

Carbonic anhydrases but also NBCe1 are expressed in various organs. A possible interaction of NBCe1 and CAII could for example take place in the kidney or brain, where an expression of both proteins has been shown (see for review: [2], [19]). CAI is expressed in the colon [56], [57] and CAIII is highly expressed in the human skeletal muscle [58], both could interact with NBCe1, which is also present in these tissues [59], [60].

In conclusion, we have shown for the first time that both intracellular isoforms, CAI and CAIII, enhance NBCe1 transport activity, similar to that of intracellular CAII, after heterologous expression in *Xenopus* oocytes. This effect is likely to be attributable to the catalytic activity of the different CA isoforms. CAIII showed robust catalytic activity in intact cells, and was therefore, similar as CAI and CAII, enhancing NBCe1 transport activity. Our results indicate that the augmenting of NBCe1 activity does not require the intramolecular proton shuttle of CAII, which is possibly rescued in the intact oocyte, just as found for the enzymatic activity of the *in vitro* less active isoforms CAI and III.
